# Zambia’s drive to eliminate malaria faces challenges

**DOI:** 10.2471/BLT.18.020518

**Published:** 2018-05-01

## Abstract

Zambia is one of eight southern African countries aiming to eliminate malaria in the next few years. Sam Loewenberg reports.

It’s 4 am and Joba Edward is sitting on a hill amongst a cluster of tin roof huts in Makuti village in the Southern Province, one of Zambia’s 10 provinces. Usually he trawls for fish. Today he is fishing for mosquitoes, and he is the human bait.

A mosquito lands on his leg. He places a rubber tube nearby and sucks the mosquito into a trap and deposits it in a lidded cup, noting the time of capture.

Later, Edward hands his catch over to the entomologists, who will measure several things, including the proportion of *Anopheles* mosquitoes infected with malaria parasites and the human biting rate.

These data will, in turn, feed into surveillance used to guide the malaria programme in the country’s Southern Province.

Edward is one of a legion of volunteers and health workers involved in a multipronged effort to eliminate malaria in Zambia.

In 2007, Bill and Melinda Gates, co-chairs of the world’s largest private foundation supporting global health programmes and research, declared eradication as the global goal in the fight against malaria. Eradication refers to wiping out a disease on a global level, while elimination refers to such efforts in a country or region.

Zambia is one of several countries that have switched from the goal of malaria control – reducing the number of cases to a very low level – to elimination, defined as reducing the number of indigenous cases to zero.

“Zambia is a wonderful example of country ownership and country leadership in the fight against malaria.”

Pedro Alonso

Supporters of the elimination agenda point to the success of the Maldives and Sri Lanka, which received World Health Organization (WHO) certification for malaria elimination in 2015 and 2016, respectively.

Unlike the two island nations, Zambia is a landlocked country that shares borders with eight countries, some of which are highly malaria endemic. Even if malaria is eliminated inside the country, public health authorities would have to continue to monitor travellers entering via multiple border crossing points to prevent imported cases sparking renewed malaria outbreaks.

Some parts of Zambia – such as the Southern Province – have made huge progress in reducing the burden of malaria, but the country of 15.9 million people has not yet achieved overall control.

While many admire Zambia’s achievements to date, some question whether the country will actually meet its ambitious goal of zero indigenous cases by 2021.

“Zambia is a wonderful example of country ownership and country leadership in the fight against malaria. The country has made incredible progress,” says Dr Pedro Alonso, the Director of the WHO Global Malaria Programme. “Whether they will actually achieve full elimination by the target date remains to be seen.”

Nationally, the reported number of deaths attributed to malaria declined by about 62% with 4834 in 2010, compared with 1827 in 2016, according to the *World malaria report 2017.*

During the same time period, however, the number of new malaria cases increased from about 4.5 million to 5.2 million. The increase may be due to population increases as well as increased service coverage allowing more cases to be detected, says Duncan Earle, Country Director of the Malaria Control and Elimination Partnership in Africa, a programme run by nongovernmental organization PATH.

In areas where there is a moderate-to-high burden of malaria transmission, with between 50 and 500 or more cases per 1000 people, the health ministry takes a three-pronged approach of mass drug administration (with fixed-dose combination treatment dihydroartemisinin-piperaquine), vector control (indoor residual spraying and distribution of insecticide-treated nets (ITN)) and case management (diagnosis and treatment).

The coverage and quality of malaria prevention and treatment has improved thanks to a large cadre of community health workers across the country.

Zambia’s malaria efforts underscore the challenges developing countries’ disease control programmes face in areas with poor infrastructure, weak health systems and porous borders. It is one of eight southern African countries, known as the E8, that have set themselves the joint 2030 goal of elimination.

Last year, two of those countries (Botswana and Namibia) had outbreaks and four (Angola, Mozambique, South Africa and Zimbabwe) had localized increases of cases. Swaziland is also in the group.

Funding gaps and growing mosquito resistance to the insecticide in ITNs further complicate matters, says Dr Elizabeth Chizema, the director of the Zambia National Malaria Elimination Centre.

Zambia’s success in moving towards malaria elimination has been geographically uneven, reflecting disparities in wealth and health system strength in different regions, uneven donor funding patterns, as well as differences in the epidemiology of the disease across the country making it easier to control malaria in some areas.

In the Southern Province where prevalence has fallen from 8% in 2012 to 0.6% in 2015, the goal of zero indigenous cases seems within reach.

Here, the malaria effort is aggressive, with active case finding. That means that if somebody comes down with malaria, community health volunteers test everyone living within a 140-metre radius of the sick person with a rapid diagnostic test that is currently being evaluated in a field trial. The volunteers also directly observe patients taking their medication for three days to make sure they take the required dose.

In the Eastern Province, the decline in malaria prevalence was from 25% to 13% between 2012 and 2015, however, it increased in seven other provinces, and remained at 32% in Luapula Province.

The north of the country is lagging behind, reflecting inequalities in investment and traditionally high levels of malaria transmission, according to Dr Chitalu Chilufya, Zambia’s health minister.

“Sometimes we have seen donor bias for certain regions, and sometimes resource allocation is not well informed by data,” Chilufya says, adding that the government is currently investing in better health information systems to give malaria efforts a robust evidence base.

For Chilufya, malaria is a drag on the economy. “Malaria directly impacts negatively on our economic development agenda,” he says, noting that the government has plans to raise additional funds for the malaria campaign by introducing taxes on alcohol, tobacco and junk food.

The more deprived parts of the country – with poor overall health indicators, greater poverty, and weak health systems and infrastructure – present more challenging environments to operate in, according to Melanie Luick-Martins, who heads USAID’s health programme, one of the few donors investing in deprived parts of Zambia.

“We must not forget the lessons of the eradication era of the 1950s and 1960s, especially the importance of country ownership, community empowerment and domestic funding.”

Richard Kamwi

Globally, the fight against malaria has stalled. According to the *World malaria report 2017,* funding for anti-malaria efforts has fallen far short of what is needed, particularly in high-burden countries. Five million more malaria cases were reported in 2016 than in 2015, while 445 000 malaria deaths were reported in 2016, hardly changed from the previous year. However, more countries are coming closer to elimination than ever before, with 44 countries in 2016 reporting fewer than 10 000 cases, compared to 37 in 2010.

In Zambia, funding needs for malaria elimination efforts are projected to be about US$ 160 million a year until 2021, but so far only about half of that has come in – two thirds from international donors and the rest from the Zambian government.

Other challenges include shortages of medicines, supplies and health workers with adequate training and supervision at the community level.

However, data from Zambia show that getting people who own ITNs to use them properly is less of a concern. A study published in 2014 in *PLOS One* reports use to be around 68%.

In contrast to other programmes, the malaria programme is well stocked with rapid diagnostic tests and medication. However, community health workers are unpaid volunteers, leading to high turnover.

While Zambia remains heavily dependent on external funding for its malaria elimination efforts, critics have questioned whether the disease can be successfully tackled without building stronger health systems first.

For Dr Richard Kamwi, the 1955–1969 Global Malaria Eradication Programme (GMEP) offers important lessons for today’s renewed effort. Kamwi is a former health minister of Namibia, who represents the E8.

The GMEP faltered with top-down approaches, an over-reliance on residual spraying with dichlorodiphenyltrichloroethane (DDT) and treatment with chloroquine. 

“We must not forget the lessons of the eradication era of the 1950s and 1960s, especially the importance of country ownership, community empowerment and domestic funding,” Kamwi says, adding: “One of the reasons why the programme failed was resistance to chloroquine and DDT. The community did not get ahead of that fast enough, programmes started to fail and funders started to pull out.”

“We spend a lot of time monitoring resistance,” says Kamwi, referring to mosquito resistance to insecticides used to treat mosquito nets. “At this point [resistance] is manageable, but going forward, we certainly need new insecticides and new vector control tools.”

The challenge of mosquito resistance to insecticides and recent evidence this may be increasing has officials worried, especially resistance to pyrethroids, the only insecticide class WHO recommends for use in ITNs.

Underscoring the need for urgent action, Unitaid, a global health financing facility, and the Global Fund to fight AIDS, Tuberculosis and Malaria, issued a joint call last year for research and development of new vector control tools.

“While we do see increasing evidence of insecticide resistance among mosquito vectors, there are, as yet, no data to show that this is compromising ITN effectiveness,” Alonso says, adding: “That said, there is work being done to identify new tools in the likelihood that resistance does begin causing operational failures.”

**Figure:**
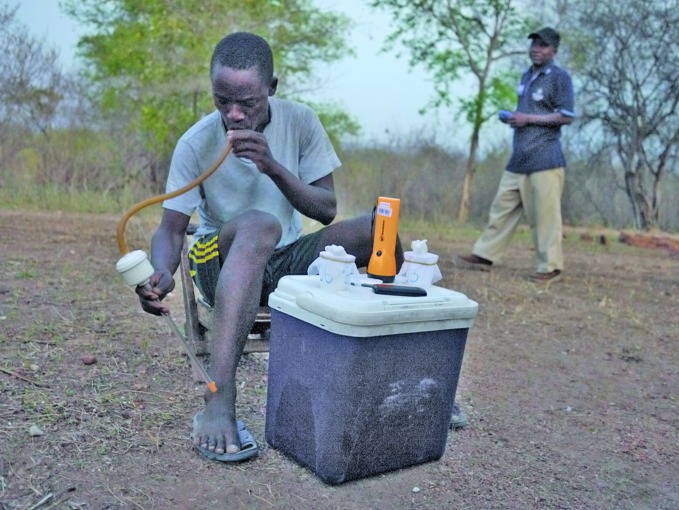
Joba Edward catching a mosquito in Zambia’s Southern Province.

WHO/S. Loewenberg

